# The N Terminus of Adhesion G Protein–Coupled Receptor GPR126/ADGRG6 as Allosteric Force Integrator

**DOI:** 10.3389/fcell.2022.873278

**Published:** 2022-06-23

**Authors:** Jakob Mitgau, Julius Franke, Camilla Schinner, Gabriele Stephan, Sandra Berndt, Dimitris G. Placantonakis, Hermann Kalwa, Volker Spindler, Caroline Wilde, Ines Liebscher

**Affiliations:** ^1^ Rudolf Schönheimer Institute for Biochemistry, Molecular Biochemistry, University of Leipzig, Leipzig, Germany; ^2^ Department of Biomedicine, University of Basel, Basel, Switzerland; ^3^ Department of Neurosurgery, Kimmel Center for Stem Cell Biology, Laura and Isaac Perlmutter Cancer Center, NYU Grossman School of Medicine, New York, NY, United States; ^4^ Rudolf-Boehm-Institute for Pharmacology and Toxicology, University of Leipzig, Leipzig, Germany

**Keywords:** adhesion GPCR, mechano-activation, signal transduction, allosteric modulator, activating antibody, extracellular matrix ligand

## Abstract

The adhesion G protein–coupled receptor (aGPCR) GPR126/ADGRG6 plays an important role in several physiological functions, such as myelination or peripheral nerve repair. This renders the receptor an attractive pharmacological target. GPR126 is a mechano-sensor that translates the binding of extracellular matrix (ECM) molecules to its N terminus into a metabotropic intracellular signal. To date, the structural requirements and the character of the forces needed for this ECM-mediated receptor activation are largely unknown. In this study, we provide this information by combining classic second-messenger detection with single-cell atomic force microscopy. We established a monoclonal antibody targeting the N terminus to stimulate GPR126 and compared it to the activation through its known ECM ligands, collagen IV and laminin 211. As each ligand uses a distinct mode of action, the N terminus can be regarded as an allosteric module that can fine-tune receptor activation in a context-specific manner.

## Introduction

The adhesion G protein–coupled receptor (aGPCR) GPR126/ADGRG6 plays an essential role in several important physiologic and pathogenic processes, including myelination (Monk et al., 2009; Monk et al., 2011; Mogha et al., 2013; Ravenscroft et al., 2015), peripheral nerve injury and repair (Mogha et al., 2016), the development of the peripheral nervous system (PNS) (Ravenscroft et al., 2015), and the differentiation of osteoblasts (Sun et al., 2020) and adipocytes (Suchý et al., 2020). Furthermore, an association of *GPR126* variants with the development of scoliosis was found in humans and mice (Kou et al., 2013; Karner et al., 2015; Xu et al., 2015; Qin et al., 2017; Kou et al., 2018; Liu et al., 2018; Xu et al., 2019a; Xu et al., 2019b; Xu et al., 2019c; Man et al., 2019; Takeda et al., 2019). Thus, pharmacological targeting of this receptor is of high interest. The physiological implications are mainly attributed to the modulation of cAMP levels by the receptor, which is achieved through the receptor’s coupling to G_s_ protein (Mogha et al., 2013).

Like other aGPCRs, GPR126 harbors an endogenous tethered agonistic sequence located distal of the GPS cleavage motif, termed the *Stachel* sequence (Liebscher et al., 2014). Synthetic peptides derived from this integral agonist can be used as agonists on the receptor (Liebscher et al., 2014; Demberg et al., 2017). More recently, small-molecule agonists have been identified (Bradley et al., 2019; Diamantopoulou et al., 2019), but similar to agonistic peptides, they lack specificity for the receptor (Demberg et al., 2017; Bradley et al., 2019). Additional activation can be achieved through the receptor’s N-terminal ligands collagen IV (Paavola et al., 2014), prion protein PrP^C^ (Küffer et al., 2016), and laminin 211 (Petersen et al., 2015). Yet, none of these agonists is specific for GPR126, and ECM proteins lack characteristics of a classic receptor agonist as they are long-lived, stable molecules with essentially no diffusivity (Bassilana et al., 2019; Baxendale et al., 2021). Thus, it is unclear how the interaction between aGPCR and the ECM can be interpreted as a specific signal to modulate receptor activity levels. Mechanical forces are suggested to facilitate this interaction (Liebscher et al., 2014; Stoveken et al., 2015; Scholz et al., 2017; Dannhäuser et al., 2020). However, the force input as well as the structural components needed for activation have not been defined.

Targeting the large N terminus of an aGPCR with an antibody provides a specific way for receptor activation, which has been successfully shown for two other representatives of this receptor class (Yona et al., 2008; Bhudia et al., 2020; Chatterjee et al., 2021). The mechanism behind this N-terminal mediated signal is currently as unclear as the signals mediated by the ECM ligands. Understanding these fundamental activation processes will not only increase our understanding of the physiological circumstances that govern GPR126-mediated functions, but it will also set the stage for allosteric pharmaceutical targeting of this (Lu and Zhang, 2019) and potentially other aGPCRs. Allosteric modulation of a GPCR is of high pharmaceutical interest as it induces intermediate activation states, which potentially form the basis for activity-specific and biased modulation of signaling pathways (Ni et al., 2020; Lu et al., 2021a; Lu et al., 2021b; Wang et al., 2022).

In the absence of a specific antibody recognizing the N terminus of GPR126, we used an antibody recognizing an N-terminal HA epitope in GPR126, which was sufficient to activate the receptor. Our study characterized the structure–function prerequisites for this antibody-mediated activation and describes in real time the type and strength of the mechanical input needed to activate GPR126 through either an antibody or endogenous ligands, collagen IV and laminin 211. We conclude that the activation through the antibody is most likely mediated through cross-linking of the receptor, while collagen IV and laminin 211 need specific pushing and pulling forces. As the occurrence of ECM molecules is timely and locally regulated in tissues, a temporal–spatial and force-specific activation of GPR126 can be achieved through the N terminus as an allosteric force integrator.

## Materials and Methods

If not stated otherwise, all standard substances were purchased from Sigma Aldrich (Taufkirchen, Germany), Merck (Darmstadt, Germany), and C. Roth GmbH (Karlsruhe, Germany). The cell culture material was obtained from Thermo Fisher Scientific (Schwerte, Germany), and primers were obtained from Microsynth Seqlab GmbH (Göttingen, Germany).

The magnetic stimulator was created by embedding N45 NdFeB magnets (https://www.supermagnete.de/data_sheet_S-08-05-N.pdf) into a custom 3D-printed holder matching the outer diameter of a corresponding cell culture plate.

### Plasmid Generation

The constructs of human GPR126 and ∆CUB, ∆PTX, and ∆CUB∆PTX mutants have been described previously (Mogha et al., 2013; Liebscher et al., 2014; Petersen et al., 2015). Point mutations for R468A and H839R constructs were inserted by quick change mutagenesis. In brief, plasmid DNA was amplified with PCR and then digested with DpnI restriction enzyme for 4 h at 37°C prior to heat shock transformation in *E. coli*. The mRuby epitope was inserted into the human GPR126 construct after the hemagglutinin (HA) epitope by a PCR-based site-directed mutagenesis and fragment replacement strategy. The sequences of all generated mutants of human GPR126 were verified by Sanger sequencing (Microsynth Seqlab, Göttingen, Germany). The GPR126/pULTRA construct was generated using one-step isothermal DNA assembly (Gibson et al., 2009). The reaction buffer was prepared according to the protocol. All enzymes were acquired from NEB: T5 exonuclease (M0363), Taq DNA Ligase (M0208), and Phusion DNA Polymerase (M0530). The WT receptor DNA for the assembly was obtained *via* PCR using the previously described construct of the full-length human GPR126 in the pcDps vector (Liebscher et al., 2014). The pULTRA [pULTRA was a gift from Malcolm Moore, Addgene plasmid #24129 (Lou et al., 2012)] vector was restriction-digested using Xba1 (NEB, R0145).

### Anti-HA Fab Fragment Generation

Sequences encoding Anti-HA Fab (clone 12CA5) heavy and light chains were codon-optimized for human cells and synthesized and cloned into pcDNA3.4 (Thermo Fisher Scientific) by Genscript. The DNA of heavy and light chains was mixed in the ratio 1:1, transfected into Expi293 cells (Thermo Fisher Scientific) using PEI Max (Polysciences), and expressed at 37°C for 7 days. Fab was purified from clarified culture supernatants using a CaptureSelect CH1-XL column (Thermo Fisher Scientific) and buffer-exchanged into Tris-buffered saline (100 mM NaCl, 20 mM Tris, pH7.5) using a HiPrep 26/10 desalting column (Cytiva, Freiburg, Germany). Binding of the Fab fragment to the present HA-tag was proven to be concentration-dependent on P2Y_12_-transfected cells, which served as receptor expression positive control (Supplementary Figure S1A).

### cAMP Accumulation Assays

The human N- and C-terminally tagged GPR126 was heterologously expressed in COS-7 cells grown in Dulbecco’s minimum essential medium (DMEM) supplemented with 10% fetal bovine serum (FBS), 100 units/ml penicillin, and 100 μg/ml streptomycin or the GripTite 293 MSR Cell Line (Thermo Fisher Scientific) (HEK-GT) grown in DMEM supplemented with 10% fetal bovine serum, 1% G418 (Thermo Fisher Scientific, 10131035) and 1% Non-Essential Amino Acids (Thermo Fisher Scientific, 11140050) at 37°C and 5% CO_2_ in a humidified atmosphere. The used variant of the mouse GPR114 (ADGRG5) is tagged just like GPR126 and has been published before (Wilde et al., 2016). All steps of the assays were performed the same way for both receptors: the cells were split into 48-well plates (3×10^4^ cells/well for COS-7 or 1.3 × 10^5^ cells/well for HEK-GT) for antibody stimulation assays or into 96-well plates (1.5 × 10^4^ cells/well for COS-7 or 4.5 × 10^4^/well for HEK-GT) for peptide stimulation. Transfection was carried out with Lipofectamine 2000 (Thermo Fisher Scientific) according to the manufacturer’s protocol using 50 ng (96-well plates) or 500 ng (48-well plates) of receptor plasmid DNA/well. 48 h after transfection, GPR126 and empty vector–transfected cells were stimulated with the indicated concentrations of anti–HA antibody (H3663, stock concentration 1 mg/ml, Sigma-Aldrich), anti-HA Fab fragment (stock concentration 1 mg/ml, kind gift from T. Schiffner), anti–HA conjugated super paramagnetic Dynabeads^®^ (14311D, stock concentration 10 mg/ml, Thermo Fisher Scientific) or anti–FLAG antibody (F1804, Sigma-Aldrich) in DMEM for 1 h, followed by incubation with 3-isobutylmethyl-xanthine (1 mM)-containing medium or including the secondary antibody anti-mouse IgG (Fc specific, M2650, Sigma-Aldrich) for 30 min. For peptide stimulation, a *Stachel*-sequence derived peptide was diluted in IBMX-containing medium. A peptide solution from purified powder was achieved by preparing a 100 mM in 100% DMSO solution, which was further diluted into 10 mM stocks using a 50 mM pH 8 Tris buffer and finally pH-controlled. Peptide concentrations used in assays are 1 mM. The 1 mM peptide solution contains 1% DMSO and 10% Tris buffer for dilution. After stimulation, the cells were lysed in LI buffer (PerkinElmer, Rodgau, Germany) and frozen at −80°C until measurement. To measure cAMP concentration, the Alpha Screen cAMP assay kit (PerkinElmer) was used according to the manufacturer’s protocol. The accumulated cAMP was measured in 384-well white OptiPlate microplates (PerkinElmer) with the EnVision Multilabel Reader (PerkinElmer). Super paramagnetic Dynabeads^®^ were conjugated with anti–HA antibody according to the manufacturer’s protocol. Briefly, Dynabeads were weighed, washed with C1 solution, mixed with an appropriate amount of anti–HA antibody in C1 solution, C2 solution was added, and the mixture was incubated overnight at 37°C on a roller, followed by washing afterward.

### Enzyme-Linked Immunosorbent Assay

The cells were split into 48-well plates (3 × 10^4^ cells/well for COS-7 or 1.3 × 10^5^ cells/well for HEK-GT). To estimate cell surface expression of receptors carrying an N-terminal HA tag, an indirect cellular enzyme-linked immunosorbent assay (ELISA) was used (Schöneberg et al., 1998). Briefly, the cells were transfected with the indicated constructs. 48 h after transfection, the cells were fixed with 4% formaldehyde, washed with PBS, blocked with 10% FBS medium, and incubated with anti–HA POD-conjugated antibody followed by *o*-phenylendiamine treatment. Optical densities were measured at a wavelength of 492 nm with the EnVision Multilabel Reader (PerkinElmer).

### Western Blot

For Western blot analysis, the cells were split into 24-well plates (6 × 10^4^ cells/well for COS-7) and transfected with either 250 ng of empty pcDps vector, GPR126, GPR126 R468A, or GPR126 R468A/H839R using the standard protocols described earlier. Every other transfected construct was incubated with the primary anti–HA antibody for an hour as explained before. Supernatants were harvested and cells were lysed with the addition of 300 μL of 2x SDS loading dye (#S3401, Sigma-Aldrich). After freeze–thaw cycling, the lysates were run on a 10% SDS-PAGE gel, followed by Western blotting. The PVDF membranes were activated using 100% methanol, and transfer was performed for 1 h at 80 V. The membranes were blocked for 1 h with 5% non-fat dry milk in TBST buffer, washed three times with TBST buffer, and incubated overnight with either primary antibody (rabbit ant-HA, #3724 and anti-GAPDH, #97166 antibody, Cell Signaling). The following day, the membranes were again washed with TBST buffer and incubated with secondary anti-rabbit antibody, which has an HRP conjugation (#7074, Cell Signaling) for 1 h at room temperature. Following three washing steps with TBST buffer, SuperSignal West Pico PLUS Chemiluminescent Substrate (Thermo Fisher Scientific) was added to membranes to visualize protein bands using the Bio-Rad Gel Doc Imager.

### AFM

For all atomic force microscopy (AFM) experiments, HEK-GT cells were cultured at the same conditions as for the *in vitro* functional assays. Cells were seeded on 24-mm glass coverslips (coated with Poly-L-Lysin (Sigma-Aldrich, P4707), (1% PLL solution incubated at 37°C for 5 min, then washed with PBS and dried under a sterile hood) in 6-well plates (1.5 × 10^6^ cells/well) and co-transfected with the cAMP sensor Pink Flamindo (Addgene plasmid #102356) and either an empty vector or the given GPR126 construct in the pULTRA vector on the next day using Lipofectamine 2000 according to the manufacturer’s protocol. The media were changed to the described culture media ∼24 h after transfection and AFM measurements took place ∼48 h after transfection: the coverslips were transferred into a 35-mm cell culture dish and washed with DMEM without phenol red three times and placed into AFM coverslip holders. 750 µL of culture media without phenol red was added, and the cells were stored at 37°C with 5% CO_2_ until right before the measurements took place.

Tipless silicon nitride AFM cantilevers (NanoWorld, PNP-TR-TL) were coated with monoclonal anti–HA antibodies produced in mouse (H3663, Sigma-Aldrich) or Fc-control for the antibody-based mechano-activation experiments using flexible PEG spacers as described before (Ebner et al., 2007). Recombinant human Fc was expressed by transfected Chinese hamster ovary (CHO) cells. The supernatant was collected and purification was carried out *via* His-tag using HisLink Protein Purification Resin (V8823, Promega) according to the manufacturer’s instructions. Protein purity was confirmed by Western blot analysis. For the ligand-based mechano-activation experiments, tipless silicon nitride AFM cantilevers were washed twice in chloroform and dried. The cantilevers were then placed into a collagen IV (C6745, Sigma Aldrich) or laminin-211 (LN221, BioLamina, Sundbyberg, Sweden) solution (0.15 mg/ml) and incubated at 4°C overnight. The next day, the cantilevers were washed in HBSS twice and stored in HBSS at 4°C until use.

AFM measurements were performed using a Nanowizard IV AFM (Bruker, Billerica, MA) mounted on an IX 83 inverted optical microscope equipped with a 63x PL APO NA 1.42 oil objective (both Olympus Life Sciences, Wallisellen, Switzerland) and coupled to an X-Cite Exacte Light source (Excelitas Technologies, Waltham, MA)*.*

Cantilevers were calibrated using the thermal noise method according to (Slattery et al., 2013).

Successfully double-transfected cells were identified by the GFP from the pULTRA vector and the Pink Flamindo fluorescence signal. The cell was then imaged three times in the following order: Brightfield, GFP, and Pink Flamindo using a Zyla sCMOS camera (Andor Technology, Belfast, Northern Ireland). The AFM cantilever was then placed centrally on the cell and approached until it contacted the cell surface. Proper positioning was verified by a brightfield image, and the baseline Pink Flamindo signal was recorded. During stimulation, all light sources except the AFM laser were turned off. Immediately after the stimulation was finished, another image of the Pink Flamindo signal was obtained using the same exposure time as before.

For force clamp measurements, the cantilevers were initially pressed onto the cell with a force of 1 nN for 5 s in order to allow antibody/ligands and the receptor to bind. The cantilever was then retracted (1 µm/s) until the desired clamp force was reached. This value was kept constant for the indicated times before the cantilever was fully retracted. The extend and retract length was 15 µm, and extend and retract speed was 5 µm/s for all experiments.

For analysis, the Pink Flamindo images taken before and after the stimulation were compared by measuring the mean intensity of a rectangular area on the stimulated cell using Fiji ImageJ (Schindelin et al., 2012). To account for variations affecting all cells independently from the stimulation, such as bleaching, a rectangular area was measured on five other cells that were not touched by the cantilever but expressed Pink Flamindo. The average of the changes in these five reference cells was subtracted from the measured change in the stimulated cell to isolate the effect the AFM cantilever stimulation has on the receptor-transfected cell (Supplementary Figure S3A).

To evaluate the mechano-independent changes of the Pink Flamindo fluorescence signal, HEK-GT cells were split into 96-well plates (4.5 × 10^4^/well) and transfected the following day, analogous to the cAMP accumulation assays described earlier. Two days after transfection, the media were removed from the wells and 40 µL of DMEM without phenol-red was added to each well. Then, the GFP signal (transfection control) as well as the Pink Flamindo fluorescence signal was imaged using a Celigo Image Cytometer (Nexcelom Bioscience). Forskolin, pGPR126, and anti–HA antibody diluted in DMEM without phenol-red were added to a final volume of 50 µL/well and the following concentrations: 10 µM forskolin, 1 µM anti-HA antibody and 1 mM pGPR126. The cells were imaged again 60 s after the addition of the respective stimulus, and the intensity of the Pink Flamindo signal before and after was compared.

### Data Analysis

Receptor expression and activation were analyzed using one- and two-way ANOVA as well as *t*-test as indicated at each figure legend. *p* values < 0.05 were considered statistically significant (*p < 0.05; **p < 0.01; ***p < 0.001). All statistical analyses were performed by GraphPad Prism version 6.00 for Windows (GraphPad Software, Inc., La Jolla, United States) or Microsoft Excel 2016 (Microsoft Corporation, Redmond, United States).

## Results

### An Antibody Targeting the N-Terminal HA Epitope can Activate GPR126

**FIGURE 1:**
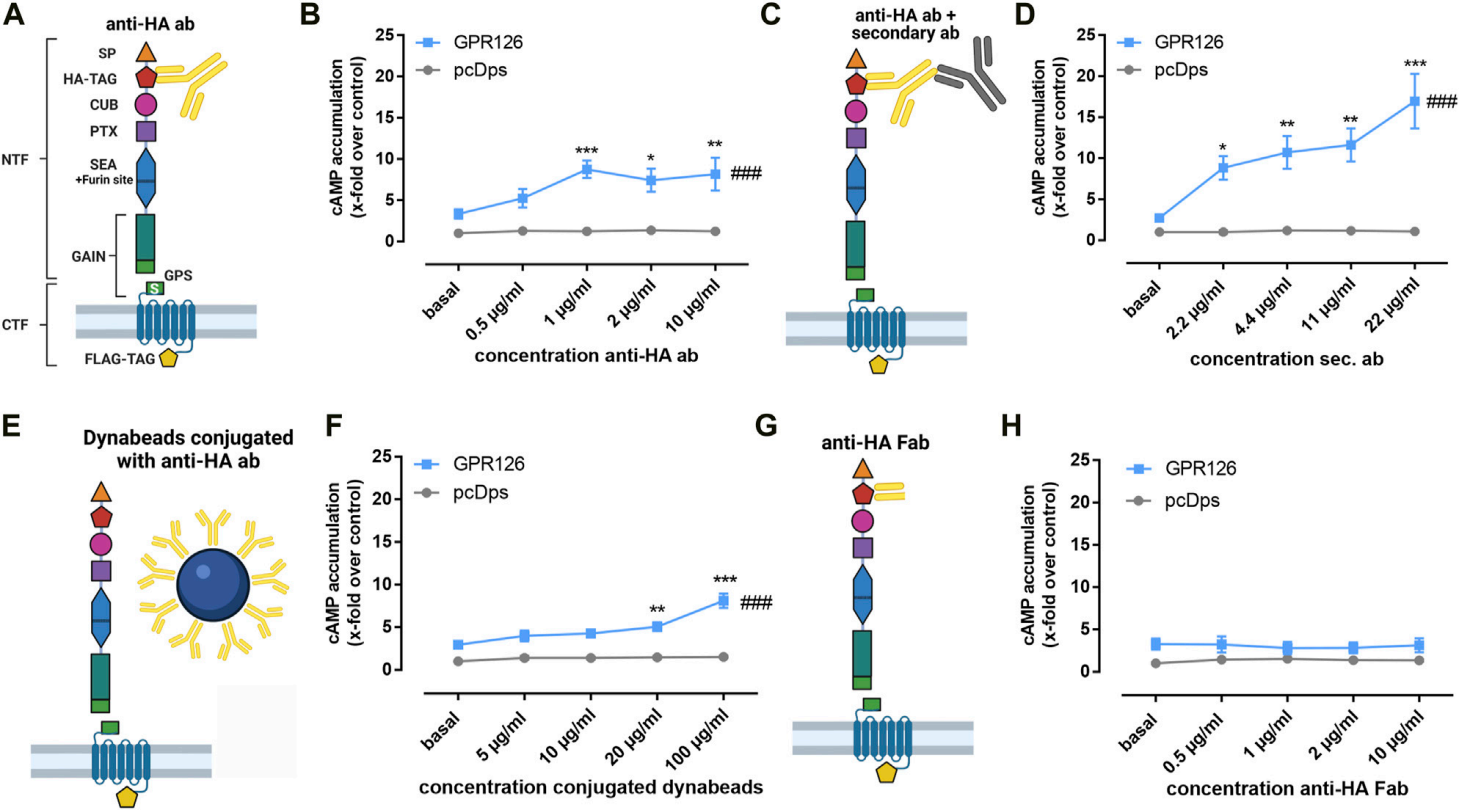
Antibodies against the N terminus activate GPR126. **(A)** Schematic setup for the anti–HA antibody (anti-HA ab) stimulation of full-length WT GPR126 in cAMP accumulation assays. The N terminus contains the signal peptide (SP, orange triangle), a complement C1r/C1s-Uegf-BMP1 domain (CUB, magenta oval), a pentraxin domain (PTX, purple square), and the sperm protein, enterokinase and agrin (SEA) domain (blue hexagon), including the furin site. The highly conserved GAIN domain (green rectangle) contains the GPS, which is followed by the *Stachel* sequence (S). In our receptor constructs, we inserted an N-terminal hemagglutinin tag (HA-TAG, red pentagon) immediately distal to the signal peptide and a C-terminal Flag tag (FLAG-TAG, yellow pentagon) right before the stop codon. **(B)**. Different concentrations of anti–HA antibody (0.5 µg/ml ≙ 3.3 µM; 1 µg/ml ≙ 6.67 µM; 2 µg/ml ≙ 13.34 µM; 10 µg/ml ≙ 66.7 µM) were used to treat vector control (pcDps) and GPR126-transfected COS-7 cells (effect of construct *p* = 0.0410, effect of concentration *p* < 0.0001, interaction construct × concentration *p* = 0.0506; two-way ANOVA). Basal cAMP level in pcDps transfected cells: 8.9 ± 0.8 nM/well. **(C,D)** Amplification of the cAMP signal of GPR126 with 1 µg/ml of anti–HA antibody and subsequent incubation with different concentrations of secondary antibody (2.2 µg/ml ≙ 14.7 µM; 4.4 µg/ml ≙ 29.3 µM; 11 µg/ml ≙ 73.3 µM; 22 µg/ml ≙ 146.7 µM) on vector control and GPR126-transfected COS-7 cells (effect of construct *p* = 0.0104, effect of concentration *p* < 0.0001, interaction construct × concentration *p* = 0.0072; two-way ANOVA). Basal cAMP level in pcDps transfected cells: 4.2 ± 1.0 nM/well. **(E,F)** Paramagnetic Dynabeads^®^ were conjugated with anti–HA ab and used in different concentrations for stimulation of vector control and GPR126-transfected COS-7 cells (effect of construct *p* = 0.0001, effect of concentration *p* < 0.0001, interaction construct × concentration *p* < 0.0001; two-way ANOVA). An empty vector (pcDps) served as a negative control. Basal cAMP level in pcDps transfected cells: 5.8 ± 0.4 nM/well. **(G,H)** cAMP accumulation upon incubation with indicated concentrations of Fab fragment (0.5 µg/ml ≙ 10 μM; 1 µg/ml ≙ 20 μM; 2 µg/ml ≙ 40 μM; 10 µg/ml ≙ 200 µM) on vector control and GPR126-transfected COS-7 cells (effect of construct *p* < 0.0001, effect of concentration *p* = 0.9920, interaction construct x concentration *p* = 0.9032; two-way ANOVA). Basal cAMP level in pcDps transfected cells: 7.7 ± 1.2 nM/well. All data are given as means ± SEM of three–five independent experiments each performed in triplicates. Statistics were performed by applying a two-way ANOVA followed by Dunnett’s post hoc analysis; *p < 0.05; **p < 0.01; ***p < 0.001. All significances given as stars (*) above individual points in the graphs show the result of the post hoc analysis, while # indicates significant concentration-dependent effects (^###^ *p* < 0.001). Corresponding raw data can be found in the repository. Schematic images were created with BioRender.com.

Since GPR126 can be activated through interaction with its extracellular ligands and mechanical stimuli, it can be assumed that the N terminus plays a decisive role in mediating these signals. However, our understanding of these dynamic processes is limited. In order to establish a specific N-terminal interacting partner of GPR126, we established an antibody-based approach. Due to the lack of antibodies targeting the endogenous GPR126 sequence, we used the hemagglutinin (HA) epitope for our experimental setup and inserted it right after the predicted signal peptide of the receptor (Figure 1A). Surprisingly, increasing concentrations of the commercially available anti–HA antibody significantly elevated cAMP levels in COS-7 cells transfected with the HA-tagged wild type (WT) GPR126 (Figure 1B) but not in the empty vector transfected control cells. In a control experiment with an anti–FLAG antibody targeting the C-terminal epitope of GPR126, no change in cAMP levels was observed (Supplementary Figure S1B).

Adding increasing concentrations of a secondary antibody to a fixed concentration of 1 µg/ml of the anti–HA antibody yielded an even stronger activation of the receptor (Figures 1C, D). We also observed the activation of GPR126 *via* the anti–HA antibody and the further modulation by the secondary antibody to be receptor-specific as we tested the experimental setup on a different mechano-sensitive aGPCR, GPR114 (ADGRG5) (Wilde et al., 2016). Neither the anti–HA antibody alone nor the addition of the secondary antibody led to any activation of the receptor (Supplementary Figure S1C). In order to elucidate whether the observed activation is a consequence of receptor cross-linking through the antibodies or due to the additional weight being attached to the receptor’s N terminus, we added anti–HA antibody–conjugated paramagnetic Dynabeads^®^, which are decisively larger in size than antibodies (Figure 1E). We observed a significant increase in cAMP levels (Figure 1F), which was comparable with anti–HA antibody–mediated activation alone (Figure 1B) but lower than the combination of primary and secondary antibody (Figure 1D), indicating that simply adding weight is not the sole key to GPR126 activation. In line with this observation, exposure to a 700-Gs magnetic field placed below the cell layer cannot further enhance conjugated Dynabead-mediated activation (Supplementary Figure S1D). Crosslinking, on the other hand, appears to be a key element to anti–HA-antibody–mediated activation as incubation with the respective monomeric Fab fragment cannot induce cAMP accumulation (Figures 1G, H).

### Anti–HA Antibody–Mediated Activation Depends on the *Stachel* Sequence and GPS Cleavage

**FIGURE 2:**
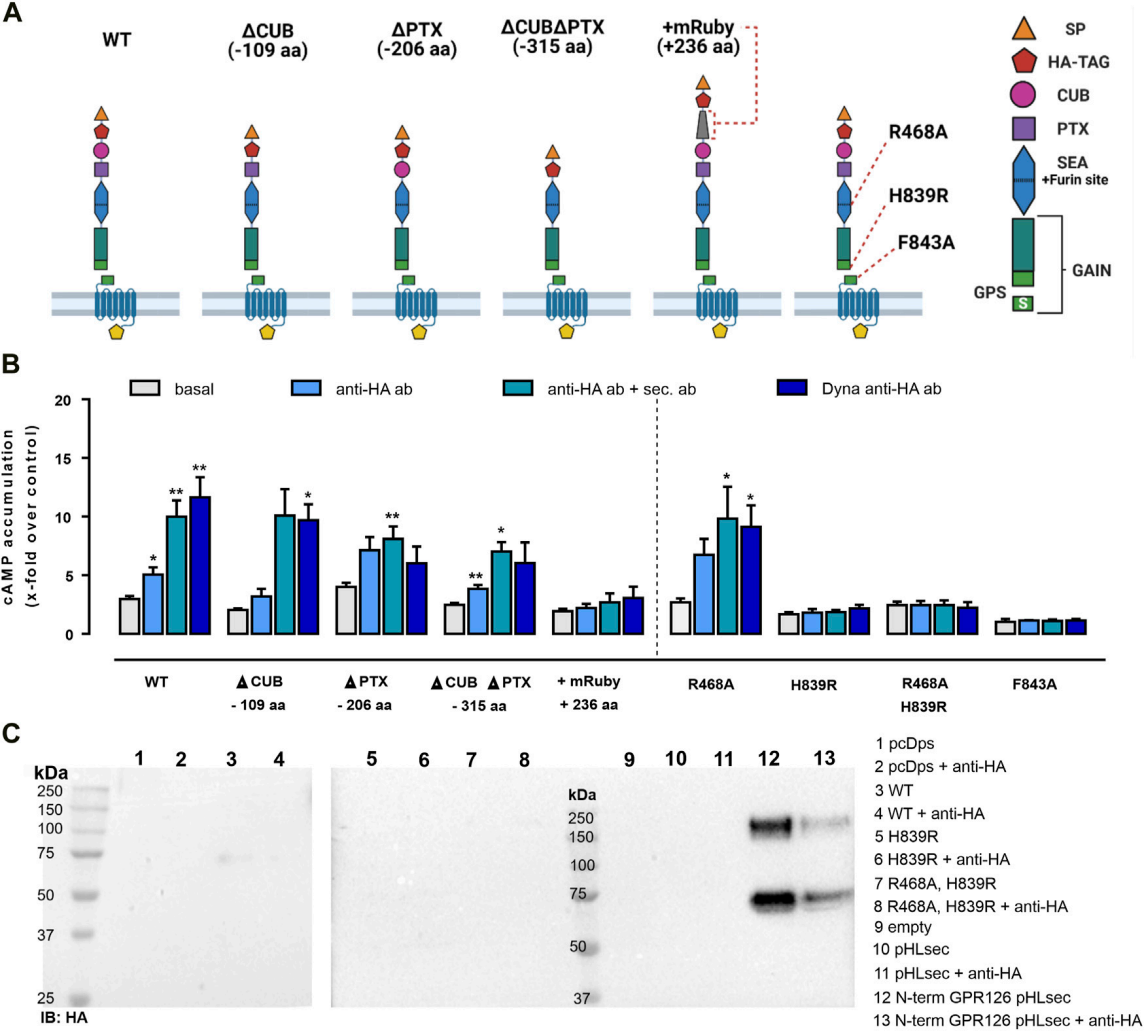
Antibody-mediated activation of GPR126 depends on autoproteolytic cleavage and is obliterated in a construct with an elongated N terminus. **(A)** Domain architecture of the human GPR126 WT and respective mutants is depicted. The positions of the furin-deficient receptor mutant R468A, the GPS cleavage mutant H839R, and the tethered agonist mutant F843A within the N terminus are displayed. Images were created with BioRender.com. **(B)** Receptor variants were transfected into COS-7 cells and analyzed with the same antibody-based approach as used in Figure 1 and tested in cAMP assays. An empty vector (pcDps) served as a negative control (cAMP level: 4.3 ± 0.7 nM/well). Data are given as mean ± SEM of three–eight different experiments each performed in triplicates. Statistics were performed by applying one-way ANOVA followed by Dunnett’s post hoc analysis; *p < 0.05; **p < 0.01; ***p < 0.001. All shown statistical significance compare the condition to the basal cAMP accumulation of the same construct. Corresponding raw data can be found in the repository. **(C)** COS-7 cells were transfected with the indicated constructs and treated with 1 µg/ml mouse anti–HA ab for 1 h, 48 h after transfection. Then, supernatants were harvested and analyzed by Western blot. Membranes were incubated with rabbit anti-HA ab and secondary HRP-conjugated anti-rabbit ab. A corresponding Western blot of cell lysates can be found in Supplementary Figure S2C. The secreted N-terminal fragment of GPR126 was only detected when the secretion vector pHLsec containing just the N-terminal fragment of GPR126 as a positive control was transfected.

The N terminus of GPR126 includes five structurally different domains (Leon et al., 2020), which can be subject to tissue-specific splicing (Knierim et al., 2019) and serve to interact with the known ligands collagen IV (Paavola et al., 2014), laminin 211 (Petersen et al., 2015), and prion protein (Küffer et al., 2016). To investigate their role in mechano-sensing, we used different N-terminal deletion mutants of GPR126, whose basal activity and expression levels were previously reported (Petersen et al., 2015). The constructs generated were ΔCUB (lacking 109 aa compared to the WT), ΔPTX (lacking 206 aa), and ΔCUBΔPTX (lacking 315 aa). In addition, we generated an N-terminal prolonged variant of GPR126 containing an mRuby-tag (236 aa prolongation compared to the WT) (Figure 2A). Reliable cell surface expression levels of the mutants were demonstrated with ELISA (Supplementary Figure S2A). The ability of receptor activation for each mutant was measured in cAMP accumulation assays using the synthetic GPR126 *Stachel* peptide as a stimulus, thereby ensuring undisturbed signaling (Supplementary Figure S2B). We stimulated these mutants with the same antibody-based setup as described in Figure 1 and found that deletion of the CUB and PTX domains yielded results highly similar to those of WT GPR126 (Figure 2B). The N-terminally prolonged mRuby construct, despite showing WT-like expression and peptide activation (Supplementary Figures S2A, B), could not be activated through antibodies (Figure 2B).

The complex architecture of the N terminus of GPR126 includes two cleavage sites; the GPCR proteolysis site (GPS) within the GAIN domain (Araç et al., 2012), at which the receptor is cleaved into an N-terminal fragment (NTF) and a C-terminal fragment (CTF) at the conserved HLT motif (Lin et al., 2004; Moriguchi et al., 2004), and a furin site, located in the SEA domain (Figure 2A). To analyze whether cleavage at either position may be required for antibody-mediated activation, we generated the furin-cleavage-deficient receptor mutant R468A, the GPS-cleavage-deficient mutant H839R, and the double-deficient mutant R468A H838R to test in antibody-mediated activation. Proper protein expression, activation capacity through *Stachel* peptide, as well as cleavage-deficiency of the mutants were confirmed (Supplementary Figure S2A–C). While mutation of the furin site (R468A) has no effect on the antibody-mediated stimulation approach, deletion of the GPS cleavage (H839R) abolishes signaling capacity similar to the previously described tethered agonist mutant F843A (Liebscher et al., 2014).

This observation could support the notion that antibody-mediated activation of this aGPCR requires the dissociation of the NTF from the CTF, as has been suggested for the activation of GPR133 (Frenster et al., 2021). To test this assumption, we harvested the supernatants from the empty vector and GPR126 transfected cells with or without anti–HA antibody stimulation and subjected them to Western blot analysis (Figure 2C). As a positive control, we used the N terminus of GPR126 encoded on the secretion vector pHLsec. No soluble NTF was found in the supernatants except for the positive control (Figure 2C). However, only a faint band was visible for the WT GPR126 of approximately 75 kDa, suggesting residual removal of the N terminus due to furin cleavage as seen for the cell lysate of the WT construct (Supplementary Figure S2C). We observed no increase in band intensities after prior stimulation with the mouse anti–HA antibody. Thus, even though the autoproteolytic procession of GPR126 at the GPS site is a prerequisite for antibody-mediated activation, it does not lead to NTF removal. It is conceivable that proteolytic processing at the GPS results in a favorable orientation of the *Stachel* sequence, which is indispensable for GPR126 activity.

### Anti–HA Antibody Activation of GPR126 Does Not Require Additional Pushing and Pulling Forces

**FIGURE 3:**
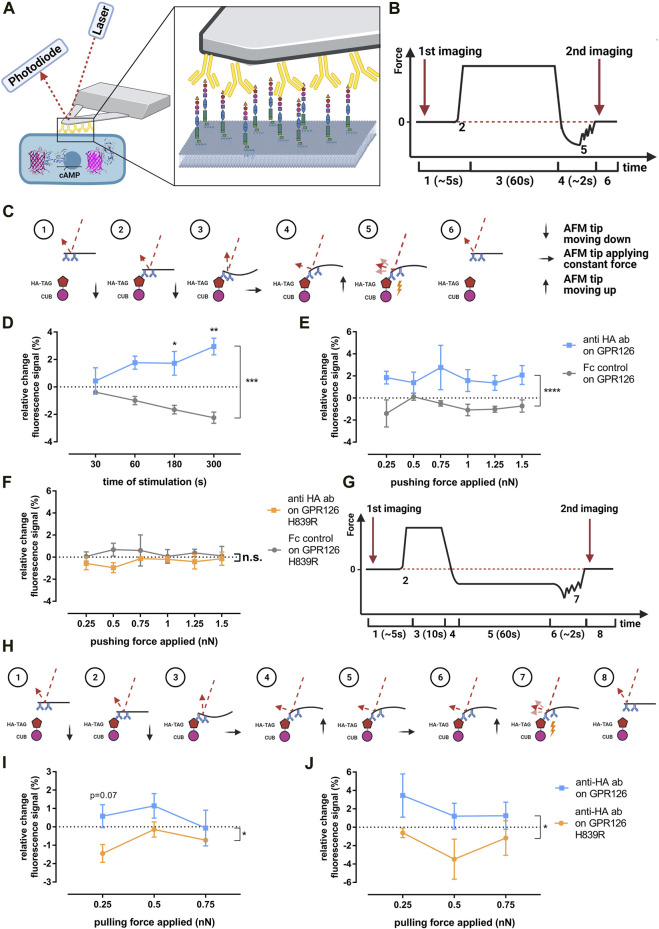
Evaluating potential mechano-activation of GPR126 *via* anti-HA antibodies. **(A)** Schematic AFM experiment setup with a coated tipless cantilever pressing on a cell co-transfected with GPR126 and the Pink Flamindo cAMP sensor. **(B,C)** Course of the AFM cantilever deflection during the pushing experiments and the forces applied to the receptor and corresponding laser deflection: (Monk et al., 2009) cantilever approaches cell; (Monk et al., 2011) point of contact between the cantilever and cell; (Mogha et al., 2013) constant pressure being applied to the cell; (Ravenscroft et al., 2015) cantilever is retracted from the cell; (Mogha et al., 2016) rupture of cantilever bindings; (Sun et al., 2020) all bindings are ruptured and the cantilever is back in its starting position. **(D)** Changes in Pink Flamindo fluorescence intensity after applying a pushing force of 1 nN on the WT receptor at different time points (effect of time *p* = 0.775, effect of coating *p* = 0.0002, interaction time × coating *p* = 0.065; two-way ANOVA). **(E,F)** Changes in Pink Flamindo fluorescence intensity after applying indicated pushing forces for 60s on the WT receptor **(E)** (effect of force applied *p* = 0.878, effect of coating *p* < 0.001, interaction force × coating *p* = 0.882; two-way ANOVA) and the H839R mutant **(F)** (effect of force applied *p* = 0.981, effect of coating *p* = 0.0867, interaction force × coating *p* = 0.924; two-way ANOVA). **(G,H)** Course of the AFM cantilever deflection during the force clamp experiments and the forces applied to the receptor and corresponding laser deflection: (Monk et al., 2009) cantilever approaches cell; (Monk et al., 2011) point of contact between cantilever and cell; (Mogha et al., 2013) initial pressure being applied to allow binding between cantilever and cell; (Ravenscroft et al., 2015) cantilever is retracted from the cell until the desired pulling force is applied; (Mogha et al., 2016) constant pulling force is applied; (Sun et al., 2020) cantilever is retracted from the cell completely; (Suchý et al., 2020) rupture of cantilever bindings; (Karner et al., 2015), all bindings are ruptured and the cantilever is back in its starting position. Changes in Pink Flamindo fluorescence intensity after applying indicated pulling forces over **(I)** 60s (effect of force applied *p* = 0.297, effect of receptor variant *p* = 0.0135, interaction force × receptor variant *p* = 0.497; two-way ANOVA) and **(J)** 300s (effect of force applied *p* = 0.405, effect of receptor variant *p* = 0.0386, interaction force × receptor variant *p* = 0.804; two-way ANOVA). Data are given as means ± SEM of three-five different experiments each measuring three individual cells for each force and time. Statistics were performed as two-way ANOVA followed by Tukey’s post hoc analysis; *p < 0.05; **p < 0.01. All significances given as stars (*) above individual points in the graphs show the result of the post hoc analysis, while # indicates significant coating-dependent **(D,E)** or receptor-variant-dependent **(I,J)** effects (^#^ *p* < 0.05; ^###^ *p* < 0.001). Corresponding raw data can be found in the repository. Schematic images **(A–C,G–H)** were created with BioRender.com. The Pink Flamindo depiction in A was taken from (Harada et al., 2017).

To evaluate whether cross-linking alone is sufficient or if additional mechanical forces are needed to activate GPR126 through the anti–HA antibody, we used an atomic force microscopy (AFM) approach. We theorized that the antibody could exert either pulling (through cross-linking) or pushing forces (through residing on the cell layer) on the receptor and that both could be quantified with AFM. To do so, tipless AFM cantilevers were coated with anti–HA antibody using PEG linkers according to a well-established protocol (Ebner et al., 2007). In contrast to cantilevers with a tip, this approach allows for multiple antibodies to bind and thus interact with the cell surface. Therefore, the mechanical force induced by the cantilever and mediated by the antibodies is applied to multiple receptors expressed on the cell surface simultaneously. The coated cantilevers were then used to apply pressure (pushing) or a force-clamp (pulling) to individual cells that were successfully co-transfected with the given receptor construct and the Pink Flamindo cAMP sensor (Harada et al., 2017) (Figure 3A). This setup allows for the simultaneous application of force and the detection of changes in intracellular cAMP levels. Single cells within a confluent monolayer were chosen for the measurements based on the detection of the GFP signal from the pULTRA vector, which allows for bicistronic expression of EGFP and GPR126 (Lou et al., 2012) and the fluorescent signal from the Pink Flamindo cAMP sensor (Harada et al., 2017) (Supplementary Figure S3A). Since COS-7 cells are not suited for AFM experiments due to their weak adherence to glass coverslips, we used GripTite™ 293 MSR (HEK-GT) cells instead. Receptor cell surface expression and activation in HEK-GT cells was confirmed prior to AFM experiments by ELISA and cAMP accumulation assays, respectively (Supplementary Figures S3B, C). Relative possible Pink Flamindo fluorescence changes upon stimulation of GPR126 with either forskolin, anti–HA antibody, or *Stachel* peptide pGPR126 are displayed in Supplementary Figure S3D.

The course of the cantilever deflection and therefore the force applied to the cell for the pushing setup is depicted in Figures 3B,C. The cAMP-evoked changes in the Pink Flamindo fluorescence were monitored by imaging right before the AFM cantilever applied pressure or a pulling force and immediately after its retraction (Figure 3B). A cantilever coated with human Fc-protein instead of anti–HA antibodies was used as a negative control.

When a pushing force of 1 nN was applied over varying times, a significant increase in the Pink Flamindo fluorescence signal could be observed in GPR126-transfected cells compared to the negative control. This effect got stronger and more significant over time, suggesting continuous stimulation of the receptor (Figure 3D). The control condition responded with a reduction of cAMP sensor intensity over time, presumably due to bleaching effects induced by the AFM detection laser. This bleaching effect was confirmed in a control experiment in which the cells were transfected with only the empty vector and Fc-protein–coated cantilevers were used to approach the cells without applying any force (Supplementary Figure S3E). Having established that a significant increase in intracellular cAMP can be achieved by pressure application *via* anti-HA antibody–coated tips, we applied varying forces over a constant time in order to quantify the strength of the pushing force needed to activate GPR126 (Figure 3E). Applying varying pushing forces (0.25–1.5 nN) with anti-HA antibody–coated tips over 60 s led to a significant increase in cAMP levels consistently over the whole range of applied forces. This indicates that either the binding or respective cross-linking of the antibody to the receptor or the pushing forces that occur during the encounter between the coated cantilever and receptor are already sufficient to activate GPR126. When we performed the same experiment with the cleavage-deficient mutant H839R (Figure 3F), which showed no activation in cAMP accumulation assays using antibodies and dynabeads (Figure 2B), we again observed no change in the cAMP-mediated Pink Flamindo signal regardless of the pressure applied.

To investigate how pulling forces affect GPR126 activation, we used the force-clamp setup as shown in Figures 3G,H. An Fc-control antibody-coated cantilever could not be used as a negative control in this case as it does not bind to the receptor or the HA-epitope and therefore no pulling forces would be applied. Instead, we compared the WT receptor to the insensitive H839R mutant, which was still able to bind the anti–HA antibody. We applied varying pulling forces (0.25–0.75 nN) over 60 s (Figure 3I) and 300 s as fold changes were very low after the shorter pulling time period (Figure 3J). There was a significant difference in the responses observed in WT and cleavage-deficient receptors. However, highest fluorescence signals were detected for the lowest pulling force (0.25 nN), while an increase in this stimulus tended to reduce cAMP production. The magnitude of the observed Pink Flamindo signal corresponds to that seen in the pushing approach. Thus, the detected increase under low pulling conditions might be due to the same reasons as the observed pushing signal (cross-linking or initial interaction push), while applying stronger pulling forces might actually inactivate the receptor.

### Endogenous Ligands of GPR126 Convey a Highly Specific Type of Mechano-Activation

**FIGURE 4:**
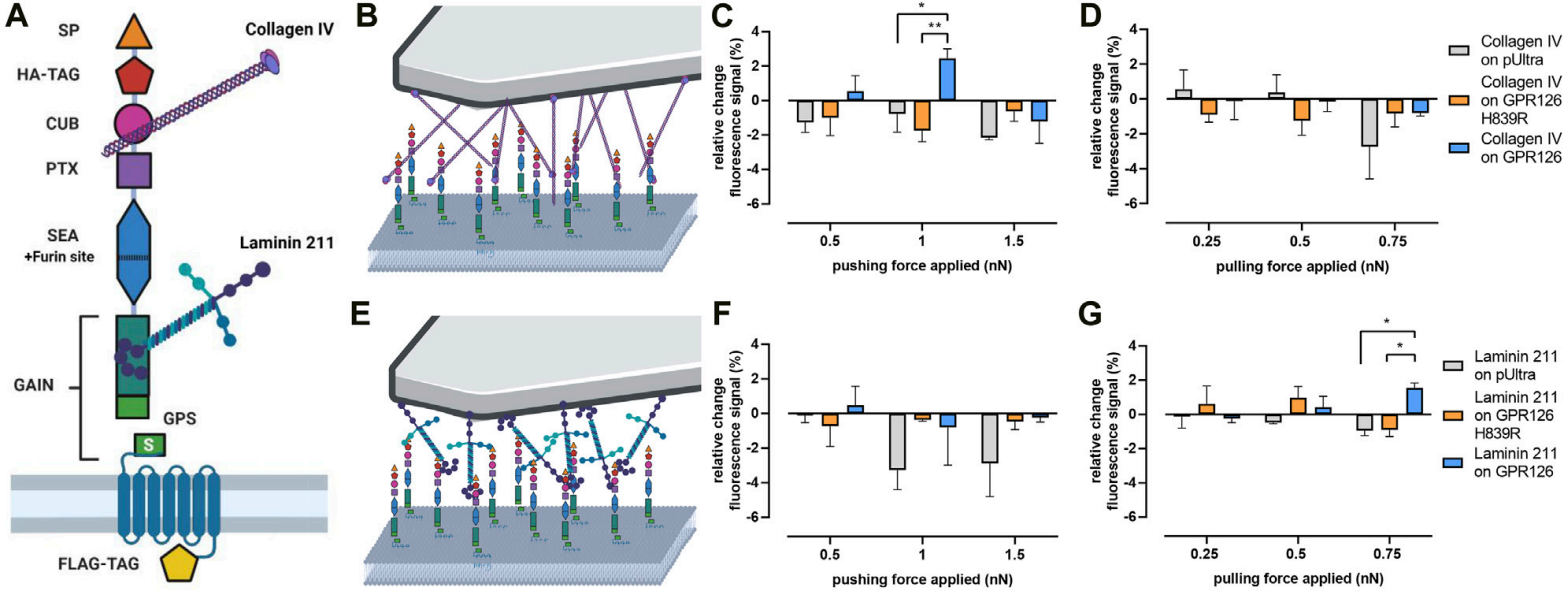
Mechano-activation of GPR126 *via* its ligands. **(A)** Domain architecture of human GPR126 with the binding sites of its ligands, collagen IV and laminin 211, is depicted. **(B,E)** Schematic demonstrating the AFM setup with **(B)** collagen IV- and **(E)** laminin 211-coated cantilevers. **(C,D)** Changes in Pink Flamindo fluorescence intensity after applying a varying pushing **(C)** or pulling **(D)** force over 60s with a collagen IV–coated cantilever. **(F,G)** Changes in Pink Flamindo fluorescence intensity after applying a pushing **(F)** or pulling **(G)** force over 60s with a laminin 211-coated cantilever. Data are given as the means ± SEM of three different experiments, each measuring three individual cells for each force. Statistics were performed as two-way ANOVA **(C)**: effect of construct *p* = 0.1452, effect of force applied *p* = 0.0635, interaction construct × force applied *p* = 0.0298; **(D)**: effect of construct *p* = 0.8096, effect of force applied *p* = 0.1748, interaction construct × force applied *p* = 0.2744; **(F)**: effect of construct *p* = 0.0537, effect of force applied *p* = 0.4536, interaction construct × force applied *p* = 0,7286; **(G)**: effect of construct *p* = 0.2225, effect of force applied *p* = 0.5884, interaction construct × force applied *p* = 0.0246) in combination with Tukey’s post hoc analysis; *p < 0.05; **p < 0.01. Significances in the graphs show the results of the post hoc analysis. Corresponding raw data can be found in the repository. Schematic images were created with BioRender.com.

We set to investigate the forces needed to stimulate GPR126 using its natural ligands. Several ligands have been shown to modulate GPR126 activity (Figure 4A): collagen IV has been described as a directly activating ligand (Paavola et al., 2014), which could be interpreted as a pushing force on the receptor as it ‘sits’ on it. Laminin 211, on the other hand, was reported to require mechanical stimuli such as shaking or vibration to induce cAMP signaling (Petersen et al., 2015), which could be a proxy for pulling forces. To test these assumptions, tipless AFM cantilevers were coated with each ligand (Figures 4B, E).

When pushing onto WT GPR126 transfected cells with a collagen IV-coated cantilever (Figure 4B), it took specifically 1 nN to induce a significant increase in cAMP levels (Figure 4C). Lower or higher pressure did not activate the receptor. Again, GPR126 H839R could not be activated through this mechanical stimulus. Applying pulling forces with collagen IV did not produce any significant changes in the Pink Flamindo fluorescence signal for neither the WT receptor nor the cleavage-deficient mutant (Figure 4D). The laminin 211-coated cantilever (Figure 4E) did not activate GPR126 through pushing (Figure 4F), but a significant increase in the Pink Flamindo fluorescence signal was seen in the force-clamp setup when applying a pulling force of 0.75 nN (Figure 4G). Lower pulling forces were not sufficient to activate the receptor.

## Discussion

The aGPCR GPR126 can be activated through different mechanisms, including agonistic *Stachel* sequence-derived peptides (Liebscher et al., 2014); its ligands collagen IV (Paavola et al., 2014), laminin 211 (Petersen et al., 2015), and prion protein (Küffer et al., 2016); the small-molecule compound apomorphine (Bradley et al., 2019); and mechanical stimuli such as vibration or shaking (Petersen et al., 2015). Yet, all of these activators lack specificity for GPR126, which hampers their practicality as tools for *in vivo* experiments or potential therapeutic approaches. The agonistic peptide pGPR126 can, for example, cross-activate the aGPCR GPR64/ADGRG2 (Demberg et al., 2017), while the ECM molecules collagen and laminin can also bind and activate integrins (Keely et al., 1995) and apomorphin is an agonist on dopamine receptors (Millan et al., 2002). In this study, we show that a commonly used monoclonal antibody targeting an N-terminal HA epitope can serve as an activator of GPR126.

Antibody-mediated GPCR activation is a known phenomenon. For example, in several disease contexts, autoantibodies target GPCRs, such as the thyroid-stimulating hormone receptor, calcium-sensing receptor, and muscarinic M1 and M2 receptors (Unal et al., 2012). Previously, the aGPCRs EMR2 and GPR56 were shown to be activated through antibodies targeting the receptor N-termini (Bhudia et al., 2020; Chatterjee et al., 2021; Yona et al., 2008). In the absence of known antibodies against GPR126, we probed a commercial anti–HA antibody targeting an artificially introduced HA epitope at the N terminus of GPR126 and found that this universal antibody was, indeed, capable of activating the receptor (Figure 1B). We, thus, wondered how the anti–HA antibody mediates this activation. For example, agonistic properties of GPCR-targeting antibodies have been previously assigned to their interaction with the cognate ligand’s binding pocket or stabilization of ligand-induced active receptor conformations (Gupta et al., 2008), for example, through cross-linking/dimerization of the receptor as has been described for β1-adrenergic (β_1_AR) receptor (Hutchings et al., 2014). With respect to the known and anticipated activation scenarios for aGPCRs, it would also be conceivable that the antibody leads to a dissociation of the NTF, resulting in exposure of the tethered agonist (Petersen et al., 2015; Mathiasen et al., 2020; Frenster et al., 2021) or that it mediates mechanical stimuli such as pushing or pulling.

Our results support the notion that the anti–HA antibody–mediated activation is most likely due to cross-linking of the receptor as a monomeric Fab fragment of the anti-HA antibody alone cannot activate GPR126 (Figure 1H). The observation that the addition of a secondary antibody further enhanced cAMP production (Figure 1D) indicated that the receptor might also respond to the weight of molecules pushing on it. However, neither the paramagnetic Dynabeads^®^ coated with the anti–HA antibody alone (Figure 1F) nor in combination with a magnet below the cell monolayer (Supplementary Figure S1B) enhanced cAMP levels compared to anti–HA antibody incubation alone. These results, as well as a lack of activation through direct pushing or pulling with an anti–HA antibody-coated cantilever in the AFM setup (Figure 3), demonstrate that cross-linking through the antibody is sufficient to activate GPR126, while no additional forces are required. Our mutagenesis data show that the anti–HA antibody–mediated stimulation of GPR126 depends on an intact tethered agonist sequence; thus, we can rule out the option that the antibody can directly interact with the endogenous agonist binding pocket (Figure 2B). Similarly, cleavage at the GPS is essential for this activation (Figure 2B), but we found no indication that this would lead to a dissociation of the NTF (Figure 2C). It can be speculated that this cleavage event would be required instead to induce a conformation that is necessary for the tethered agonist to reach its binding pocket. This is in contrast to other aGPCRs such as GPR56 (Chatterjee et al., 2021) and latrophilin (Scholz et al., 2015), whose antibody- and mechano-mediated activations, respectively, are not affected by mutations of their GPS cleavage motifs. Thus, it seems that autoproteolysis at the GPS serves distinct purposes among different receptors.

When studying aGPCR activation by ligands or antibodies, splice variants normally have to be considered since the complex exon–intron composition results in a large subset of functionally divergent receptors. As an example, activation of GPR56 through an antibody targeting the GAIN domain is dependent on a serine–threonine–proline-rich (STP) region that otherwise does not influence basal signaling levels of the receptor (Chatterjee et al., 2021). In the case of GPR126, alternative splicing influences the domain composition of the N terminus (Knierim et al., 2019). Within the N terminus, the CUB/PTX domain is of functional relevance as it serves as a point of interaction for collagen IV (Paavola et al., 2014); yet, for anti–HA antibody–mediated activation, it appears to be neglectable. In contrast, elongating the receptor’s N terminus through the addition of a fluorescent protein abolishes this activation. Similar observations have been made for the adhesion GPCR dCIRL, where elongation of the N terminus reduces the response to mechanical stimuli (Scholz et al., 2017). Thus, large artificial N-terminal domains influence signaling properties, even when they do not act as binding sites for the activating antibodies. It is currently unknown whether this is due to the simple change in length or an altered three-dimensional structure of the N terminus.

The endogenous interaction partners collagen IV and laminin 211 also bind to the N terminus of GPR126 but show different activation mechanisms. While incubation with collagen IV directly activates the receptor (Paavola et al., 2014), laminin 211 only induces cAMP production in combination with mechanical forces (Petersen et al., 2015). As the quality and the quantity of the required forces have not been defined at a single-cell level, it was hard to judge whether the *in vitro* findings could possibly be relevant in an *in vivo* context. Using the AFM approach, we found that laminin 211 requires increasing pulling forces of at least 0.75 nN (Figure 4G), while collagen IV only raises cAMP levels upon a pushing force of 1 nN (Figure 4C). Both mechano-stimulations require a cleavable GPR126. The activation patterns fit the physiological setting for these ligands in the process of myelination (Bunge et al., 1990; Paavola et al., 2014; Petersen et al., 2015), and the detected forces needed to induce a ligand-specific response are within the physiologic force range. They are below the traction forces that are normally transmitted by cell adhesions to the surrounding ECM (1–10 nN) (Balaban et al., 2001), indicating that already small changes can be detected by an aGPCR.

In summary, we were able to define the different ways in which a subset of structurally divergent molecules bound to the N terminus of GPR126 can modify the activity of this receptor. This establishes the N terminus as an allosteric integrator of signals coming from the immediate extracellular environment that is able to induce a spatio-temporal-force-dependent signal. It should, therefore, be considered a prime target for future pharmaceutical interventions as it provides the basis for receptor and potentially signaling-specific modulation of GPR126 activity.

## Data Availability

The datasets generated for this study (x-folds as well as raw values of cAMP accumulation and fluorescence changes) can be found online in “zenodo,” https://zenodo.org/record/6489015 (DOI: 10.5281/zenodo.6489015).

## References

[B1] Araç D. Boucard A. A. Bolliger M. F. Nguyen J. Soltis S. M. Südhof T. C. (2012). A Novel Evolutionarily Conserved Domain of Cell-Adhesion GPCRs Mediates Autoproteolysis. EMBO J. 31, 1364–1378. 10.1038/emboj.2012.26 22333914 PMC3321182

[B2] Balaban N. Q. Schwarz U. S. Riveline D. Goichberg P. Tzur G. Sabanay I. (2001). Force and Focal Adhesion Assembly: a Close Relationship Studied Using Elastic Micropatterned Substrates. Nat. Cell Biol. 3, 466–472. 10.1038/35074532 11331874

[B3] Bassilana F. Nash M. Ludwig M.-G. (2019). Adhesion G Protein-Coupled Receptors: Opportunities for Drug Discovery. Nat. Rev. Drug Discov. 18, 869–884. 10.1038/s41573-019-0039-y 31462748

[B4] Baxendale S. Asad A. Shahidan N. O. Wiggin G. R. Whitfield T. T. (2021). The Adhesion GPCR Adgrg6 (Gpr126): Insights from the Zebrafish Model. Genesis 59, e23417. 10.1002/dvg.23417 33735533 PMC11475505

[B5] Bhudia N. Desai S. King N. Ancellin N. Grillot D. Barnes A. A. (2020). G Protein-Coupling of Adhesion GPCRs ADGRE2/EMR2 and ADGRE5/CD97, and Activation of G Protein Signalling by an Anti-EMR2 Antibody. Sci. Rep. 10, 1004. 10.1038/s41598-020-57989-6 31969668 PMC6976652

[B6] Bradley E. C. Cunningham R. L. Wilde C. Morgan R. K. Klug E. A. Letcher S. M. (2019). *In Vivo* identification of Small Molecules Mediating Gpr126/Adgrg6 Signaling during Schwann Cell Development. Ann. N.Y. Acad. Sci. 1456, 44–63. 10.1111/nyas.14233 31529518 PMC7189964

[B7] Bunge M. B. Clark M. B. Dean A. C. Eldridge C. F. Bunge R. P. (1990). Schwann Cell Function Depends upon Axonal Signals and Basal Lamina Components. Ann. N. Y. Acad. Sci. 580, 281–287. 10.1111/j.1749-6632.1990.tb17937.x 2337301

[B8] Chatterjee T. Zhang S. Posey T. A. Jacob J. Wu L. Yu W. (2021). Anti-GPR56 Monoclonal Antibody Potentiates GPR56-Mediated Src-Fak Signaling to Modulate Cell Adhesion. J. Biol. Chem. 296, 100261. 10.1016/j.jbc.2021.100261 33837725 PMC7948743

[B9] Dannhäuser S. Lux T. J. Hu C. Selcho M. Chen J. T.-C. Ehmann N. (2020). Antinociceptive Modulation by the Adhesion GPCR CIRL Promotes Mechanosensory Signal Discrimination. Elife 9, 2020. 10.7554/eLife.56738 PMC7546736 32996461

[B10] Demberg L. M. Winkler J. Wilde C. Simon K.-U. Schön J. Rothemund S. (2017). Activation of Adhesion G Protein-Coupled Receptors. J. Biol. Chem. 292, 4383–4394. 10.1074/jbc.M116.763656 28154189 PMC5377759

[B11] Diamantopoulou E. Baxendale S. de la Vega de León A. Asad A. Holdsworth C. J. Abbas L. (2019). Identification of Compounds that Rescue Otic and Myelination Defects in the Zebrafish Adgrg6 (Gpr126) Mutant. Elife 8. 10.7554/eLife.44889 PMC6598766 31180326

[B12] Ebner A. Wildling L. Kamruzzahan A. S. M. Rankl C. Wruss J. Hahn C. D. (2007). A New, Simple Method for Linking of Antibodies to Atomic Force Microscopy Tips. Bioconjugate Chem. 18, 1176–1184. 10.1021/bc070030s 17516625

[B13] Frenster J. D. Stephan G. Ravn-Boess N. Bready D. Wilcox J. Kieslich B. (2021). Functional Impact of Intramolecular Cleavage and Dissociation of Adhesion G Protein-Coupled Receptor GPR133 (ADGRD1) on Canonical Signaling. J. Biol. Chem. 296, 100798. 10.1016/j.jbc.2021.100798 34022221 PMC8215292

[B14] Gibson D. G. Young L. Chuang R.-Y. Venter J. C. Hutchison C. A. Smith H. O. (2009). Enzymatic Assembly of DNA Molecules up to Several Hundred Kilobases. Nat. Methods 6, 343–345. 10.1038/nmeth.1318 19363495

[B15] Gupta A. Heimann A. Gomes I. Devi L. (2008). Antibodies against G-Protein Coupled Receptors: Novel Uses in Screening and Drug Development. Cchts 11, 463–467. 10.2174/138620708784911465 PMC3125642 18673273

[B16] Harada K. Ito M. Wang X. Tanaka M. Wongso D. Konno A. (2017). Red Fluorescent Protein-Based cAMP Indicator Applicable to Optogenetics and *In Vivo* Imaging. Sci. Rep. 7, 7351. 10.1038/s41598-017-07820-6 28779099 PMC5544736

[B17] Hutchings C. J. Cseke G. Osborne G. Woolard J. Zhukov A. Koglin M. (2014). Monoclonal Anti-β1-adrenergic Receptor Antibodies Activate G Protein Signaling in the Absence of β-arrestin Recruitment. MAbs 6, 246–261. 10.4161/mabs.27226 24253107 PMC3929447

[B18] Karner C. M. Long F. Solnica-Krezel L. Monk K. R. Gray R. S. (2015). Gpr126/Adgrg6deletion in Cartilage Models Idiopathic Scoliosis and Pectus Excavatum in Mice. Hum. Mol. Genet. 24, 4365–4373. 10.1093/hmg/ddv170 25954032 PMC4492399

[B19] Keely P. J. Wu J. E. Santoro S. A. (1995). The Spatial and Temporal Expression of the α2β1 Integrin and its Ligands, Collagen I, Collagen IV, and Laminin, Suggest Important Roles in Mouse Mammary Morphogenesis. Differentiation 59, 1–13. 10.1046/j.1432-0436.1995.5910001.x 7589890

[B20] Knierim A. B. Röthe J. Çakir M. V. Lede V. Wilde C. Liebscher I. (2019). Genetic Basis of Functional Variability in Adhesion G Protein-Coupled Receptors. Sci. Rep. 9, 11036. 10.1038/s41598-019-46265-x 31363148 PMC6667449

[B21] Kou I. Takahashi Y. Johnson T. A. Takahashi A. Guo L. Dai J. (2013). Genetic Variants in GPR126 Are Associated with Adolescent Idiopathic Scoliosis. Nat. Genet. 45, 676–679. 10.1038/ng.2639 23666238

[B22] Kou I. Watanabe K. Watanabe K. Takahashi Y. Momozawa Y. Khanshour A. (2018). A Multi-Ethnic Meta-Analysis Confirms the Association of Rs6570507 with Adolescent Idiopathic Scoliosis. Sci. Rep. 8, 11575. 10.1038/s41598-018-29011-7 30069010 PMC6070519

[B23] Küffer A. Lakkaraju A. K. K. Mogha A. Petersen S. C. Airich K. Doucerain C. (2016). The Prion Protein Is an Agonistic Ligand of the G Protein-Coupled Receptor Adgrg6. Nature 536, 464–468. 10.1038/nature19312 27501152 PMC5499706

[B24] Leon K. Cunningham R. L. Riback J. A. Feldman E. Li J. Sosnick T. R. (2020). Structural Basis for Adhesion G Protein-Coupled Receptor Gpr126 Function. Nat. Commun. 11, 194. 10.1038/s41467-019-14040-1 31924782 PMC6954182

[B25] Liebscher I. Schön J. Petersen S. C. Fischer L. Auerbach N. Demberg L. M. (2014). A Tethered Agonist within the Ectodomain Activates the Adhesion G Protein-Coupled Receptors GPR126 and GPR133. Cell Rep. 9, 2018–2026. 10.1016/j.celrep.2014.11.036 25533341 PMC4277498

[B26] Lin H.-H. Chang G.-W. Davies J. Q. Stacey M. Harris J. Gordon S. (2004). Autocatalytic Cleavage of the EMR2 Receptor Occurs at a Conserved G Protein-Coupled Receptor Proteolytic Site Motif. J. Biol. Chem. 279, 31823–31832. 10.1074/jbc.M402974200 15150276

[B27] Liu G. Liu S. Lin M. Li X. Chen W. Zuo Y. (2018). Genetic Polymorphisms of GPR126 Are Functionally Associated with PUMC Classifications of Adolescent Idiopathic Scoliosis in a Northern Han Population. J. Cell. Mol. Med. 22, 1964–1971. 10.1111/jcmm.13486 29363878 PMC5824397

[B28] Lou E. Fujisawa S. Morozov A. Barlas A. Romin Y. Dogan Y. (2012). Tunneling Nanotubes Provide a Unique Conduit for Intercellular Transfer of Cellular Contents in Human Malignant Pleural Mesothelioma. PLoS ONE 7, e33093. 10.1371/journal.pone.0033093 22427958 PMC3302868

[B29] Lu S. Chen Y. Wei J. Zhao M. Ni D. He X. (2021). Mechanism of Allosteric Activation of SIRT6 Revealed by the Action of Rationally Designed Activators. Acta Pharm. Sin. B 11, 1355–1361. 10.1016/j.apsb.2020.09.010 34094839 PMC8148055

[B30] Lu S. He X. Yang Z. Chai Z. Zhou S. Wang J. (2021). Activation Pathway of a G Protein-Coupled Receptor Uncovers Conformational Intermediates as Targets for Allosteric Drug Design. Nat. Commun. 12, 4721. 10.1038/s41467-021-25020-9 34354057 PMC8342441

[B31] Lu S. Zhang J. (2019). Small Molecule Allosteric Modulators of G-Protein-Coupled Receptors: Drug-Target Interactions. J. Med. Chem. 62, 24–45. 10.1021/acs.jmedchem.7b01844 29457894

[B32] Man G. C.-W. Tang N. L.-S. Chan T. F. Lam T. P. Li J. W. Ng B. K.-W. (2019). Replication Study for the Association of GWAS-Associated Loci with Adolescent Idiopathic Scoliosis Susceptibility and Curve Progression in a Chinese Population. Spine 44, 464–471. 10.1097/BRS.0000000000002866 30234802

[B33] Mathiasen S. Palmisano T. Perry N. A. Stoveken H. M. Vizurraga A. McEwen D. P. (2020). G12/13 Is Activated by Acute Tethered Agonist Exposure in the Adhesion GPCR ADGRL3. Nat. Chem. Biol. 16, 1343–1350. 10.1038/s41589-020-0617-7 32778842 PMC7990041

[B34] Millan M. J. Maiofiss L. Cussac D. Audinot V. Boutin J.-A. Newman-Tancredi A. (2002). Differential Actions of Antiparkinson Agents at Multiple Classes of Monoaminergic Receptor. I. A Multivariate Analysis of the Binding Profiles of 14 Drugs at 21 Native and Cloned Human Receptor Subtypes. J. Pharmacol. Exp. Ther. 303, 791–804. 10.1124/jpet.102.039867 12388666

[B35] Mogha A. Benesh A. E. Patra C. Engel F. B. Schöneberg T. Liebscher I. (2013). Gpr126 Functions in Schwann Cells to Control Differentiation and Myelination via G-Protein Activation. J. Neurosci. 33, 17976–17985. 10.1523/JNEUROSCI.1809-13.2013 24227709 PMC3828454

[B36] Mogha A. Harty B. L. Carlin D. Joseph J. Sanchez N. E. Suter U. (2016). Gpr126/Adgrg6 Has Schwann Cell Autonomous and Nonautonomous Functions in Peripheral Nerve Injury and Repair. J. Neurosci. 36, 12351–12367. 10.1523/JNEUROSCI.3854-15.2016 27927955 PMC5148226

[B37] Monk K. R. Naylor S. G. Glenn T. D. Mercurio S. Perlin J. R. Dominguez C. (2009). A G Protein-Coupled Receptor Is Essential for Schwann Cells to Initiate Myelination. Science 325, 1402–1405. 10.1126/science.1173474 19745155 PMC2856697

[B38] Monk K. R. Oshima K. Jörs S. Heller S. Talbot W. S. (2011). Gpr126 Is Essential for Peripheral Nerve Development and Myelination in Mammals. Development 138, 2673–2680. 10.1242/dev.062224 21613327 PMC3109596

[B39] Moriguchi T. Haraguchi K. Ueda N. Okada M. Furuya T. Akiyama T. (2004). DREG, a Developmentally Regulated G Protein-Coupled Receptor Containing Two Conserved Proteolytic Cleavage Sites. Genes cells. 9, 549–560. 10.1111/j.1356-9597.2004.00743.x 15189448

[B40] Ni D. Wei J. He X. Rehman A. U. Li X. Qiu Y. (2020). Discovery of Cryptic Allosteric Sites Using Reversed Allosteric Communication by a Combined Computational and Experimental Strategy. Chem. Sci. 12, 464–476. 10.1039/d0sc05131d 34163609 PMC8178949

[B41] Paavola K. J. Sidik H. Zuchero J. B. Eckart M. Talbot W. S. (2014). Type IV Collagen Is an Activating Ligand for the Adhesion G Protein-Coupled Receptor GPR126. Sci. Signal. 7, ra76. 10.1126/scisignal.2005347 25118328 PMC4159047

[B42] Petersen S. C. Luo R. Liebscher I. Giera S. Jeong S.-J. Mogha A. (2015). The Adhesion GPCR GPR126 Has Distinct, Domain-dependent Functions in Schwann Cell Development Mediated by Interaction with Laminin-211. Neuron 85, 755–769. 10.1016/j.neuron.2014.12.057 25695270 PMC4335265

[B43] Qin X. Xu L. Xia C. Zhu W. Sun W. Liu Z. (2017). Genetic Variant of GPR126 Gene Is Functionally Associated with Adolescent Idiopathic Scoliosis in Chinese Population. Spine 42, E1098–E1103. 10.1097/BRS.0000000000002123 28198779

[B44] Ravenscroft G. Nolent F. Rajagopalan S. Meireles A. M. Paavola K. J. Gaillard D. (2015). Mutations of GPR126 Are Responsible for Severe Arthrogryposis Multiplex Congenita. Am. J. Hum. Genet. 96, 955–961. 10.1016/j.ajhg.2015.04.014 26004201 PMC4457946

[B45] Schindelin J. Arganda-Carreras I. Frise E. Kaynig V. Longair M. Pietzsch T. (2012). Fiji: an Open-Source Platform for Biological-Image Analysis. Nat. Methods 9, 676–682. 10.1038/nmeth.2019 22743772 PMC3855844

[B46] Scholz N. Gehring J. Guan C. Ljaschenko D. Fischer R. Lakshmanan V. (2015). The Adhesion GPCR Latrophilin/CIRL Shapes Mechanosensation. Cell Rep. 11, 866–874. 10.1016/j.celrep.2015.04.008 25937282

[B47] Scholz N. Guan C. Nieberler M. Grotemeyer A. Maiellaro I. Gao S. (2017). Mechano-dependent Signaling by Latrophilin/CIRL Quenches cAMP in Proprioceptive Neurons. Elife 6. 10.7554/eLife.28360 PMC5548486 28784204

[B48] Schöneberg T. Schulz A. Biebermann H. Grüters A. Grimm T. Hübschmann K. (1998). V2 Vasopressin Receptor Dysfunction in Nephrogenic Diabetes Insipidus Caused by Different Molecular Mechanisms. Hum. Mutat. 12, 196–205. 10.1002/(sici)1098-1004(1998)12:3<196::aid-humu7>3.0.co;2-f 9711877

[B49] Slattery A. D. Blanch A. J. Quinton J. S. Gibson C. T. (2013). Accurate Measurement of Atomic Force Microscope Cantilever Deflection Excluding Tip-Surface Contact with Application to Force Calibration. Ultramicroscopy 131, 46–55. 10.1016/j.ultramic.2013.03.009 23685172

[B50] Stoveken H. M. Hajduczok A. G. Xu L. Tall G. G. (2015). Adhesion G Protein-Coupled Receptors Are Activated by Exposure of a Cryptic Tethered Agonist. Proc. Natl. Acad. Sci. U.S.A. 112, 6194–6199. 10.1073/pnas.1421785112 25918380 PMC4434738

[B51] Suchý T. Zieschang C. Popkova Y. Kaczmarek I. Weiner J. Liebing A.-D. (2020). The Repertoire of Adhesion G Protein-Coupled Receptors in Adipocytes and Their Functional Relevance. Int. J. Obes. 44, 2124–2136. 10.1038/s41366-020-0570-2 PMC7508673 32203115

[B52] Sun P. He L. Jia K. Yue Z. Li S. Jin Y. (2020). Regulation of Body Length and Bone Mass by Gpr126/Adgrg6. Sci. Adv. 6, eaaz0368. 10.1126/sciadv.aaz0368 32219165 PMC7083604

[B53] Takeda K. Kou I. Hosogane N. Otomo N. Yagi M. Kaneko S. (2019). Association of Susceptibility Genes for Adolescent Idiopathic Scoliosis and Intervertebral Disc Degeneration with Adult Spinal Deformity. Spine 44, 1623–1629. 10.1097/BRS.0000000000003179 31365516

[B54] Unal H. Jagannathan R. Karnik S. S. (2012). Mechanism of GPCR-Directed Autoantibodies in Diseases. Adv. Exp. Med. Biol. 749, 187–199. 10.1007/978-1-4614-3381-1_13 22695846 PMC12883053

[B55] Wang Y. Li M. Liang W. Shi X. Fan J. Kong R. (2022). Delineating the Activation Mechanism and Conformational Landscape of a Class B G Protein-Coupled Receptor Glucagon Receptor. Comput. Struct. Biotechnol. J. 20, 628–639. 10.1016/j.csbj.2022.01.015 35140883 PMC8801358

[B56] Wilde C. Fischer L. Lede V. Kirchberger J. Rothemund S. Schöneberg T. (2016). The Constitutive Activity of the Adhesion GPCR GPR114/ADGRG5 Is Mediated by its Tethered Agonist. FASEB J. 30, 666–673. 10.1096/fj.15-276220 26499266

[B57] Xu E. Lin T. Jiang H. Ji Z. Shao W. Meng Y. (2019). Asymmetric Expression of GPR126 in the Convex/concave Side of the Spine Is Associated with Spinal Skeletal Malformation in Adolescent Idiopathic Scoliosis Population. Eur. Spine J. 28, 1977–1986. 10.1007/s00586-019-06001-5 31079250

[B58] Xu E. Shao W. Jiang H. Lin T. Gao R. Zhou X. (2019). A Genetic Variant in GPR126 Causing a Decreased Inclusion of Exon 6 Is Associated with Cartilage Development in Adolescent Idiopathic Scoliosis Population. BioMed Res. Int. 2019, 1–8. 10.1155/2019/4678969 PMC6388357 30886859

[B59] Xu J.-F. Yang G.-h. Pan X.-H. Zhang S.-J. Zhao C. Qiu B.-S. (2015). Association of GPR126 Gene Polymorphism with Adolescent Idiopathic Scoliosis in Chinese Populations. Genomics 105, 101–107. 10.1016/j.ygeno.2014.11.009 25479386

[B60] Xu L. Wu Z. Xia C. Tang N. Cheng J. C. Y. Qiu Y. (2019). A Genetic Predictive Model Estimating the Risk of Developing Adolescent Idiopathic Scoliosis. Cg 20, 246–251. 10.2174/1389202920666190730132411 PMC6983957 32030084

[B61] Yona S. Lin H. H. Dri P. Davies J. Q. Hayhoe R. P. G. Lewis S. M. (2008). Ligation of the adhesion‐GPCR EMR2 Regulates Human Neutrophil Function. FASEB J. 22, 741–751. 10.1096/fj.07-9435com 17928360

